# The Assessment of Indoor Formaldehyde and Bioaerosol Removal by Using Negative Discharge Electrostatic Air Cleaners

**DOI:** 10.3390/ijerph19127209

**Published:** 2022-06-12

**Authors:** Chao-Yun Liu, Chao-Heng Tseng, Kai-Feng Wang

**Affiliations:** 1 Institute of Environmental Engineering and Management, National Taipei University of Technology, Taipei 106344, Taiwan; t103609005@ntut.edu.tw; 2 Union Professional Group of Architecture, Taipei 110057, Taiwan; max-typhoon@hciaq.tw

**Keywords:** electrostatic precipitator air cleaner, suspended particulates, bioaerosol, formaldehyde, indoor air quality

## Abstract

This study investigated the single-pass performance of a negative corona electrostatic precipitators (ESP) in removing suspended particulates (PM_2.5_ and PM_10_), formaldehyde (HCHO), and bioaerosols (bacteria and fungi) and measured the ozone (O_3_) concentration generated by ESP. The experimental results revealed that if the operational conditions for the ESP were set to high voltage (−10.5 kV) and low air flow rate (2.4 m^3^/min), ESP had optimal air pollutant removal efficiency. In the laboratory system, its PM_2.5_ and PM_10_ removal rates both reached 99% at optimal conditions, and its HCHO removal rate was 55%. In field tests, its PM_2.5_, PM_10_, HCHO, bacteria, and fungi removal rates reached 89%, 90%, 46%, 69%, and 85% respectively. The ESP in the laboratory system (−10.5 kV and 2.4 m^3^/min) generated 7.374 ppm of O_3_ under optimal conditions. Under the same operational conditions, O_3_ generated by ESP in the food waste storage room and the meeting room were 1.347 ppm and 1.749 ppm, respectively. The removal of HCHO and bioaerosols was primarily attributed to their destruction in the corona, as well as ozone oxidation, and collection on the dust collection plate.

## 1. Introduction

Electrostatic precipitator (ESP) air cleaners are widely used to reduce industrial and general indoor air pollution. They have a suspended particulate removal efficiency of 99% [1,2,3,4]. For ultrafine particles with a particle diameter smaller than 0.3 µm, the removal rate ranges 60–99% [5,6,7]. In an ESP, gaseous air pollutants and bioaerosols (bacteria and fungi) are removed by using a high-voltage discharge to charge suspended particulates; these particulates are then collected using dust collection plates. The collection rate increases as the discharge voltage increases [8,9,10]. During the ESP discharge process, the air surrounding the discharge electrode can form a plasma, which destroys gaseous air pollutants [11,12,13]. Han et al. (2017) [14] studied bioaerosol removal efficiency at ESP and discovered that, during the charging process, the removal amount of charged bioaerosols was proportional to the square of particle diameter. As the particle diameter increased, the removal efficiency increased, achieving 70%. Kim et al. (2018) [15] developed a new ESP with a gaseous pollutant (acetic acid, acetaldehyde, and ammonia) removal rate of 58–98%. They found that, if the air flow speed passing through the ESP was low, the pollutants stayed in the corona area for a longer time, resulting an increase in the removal rate. The corona discharge of ESP has favorable HCHO removal efficiencies [16]. Yuan et al. (2020) [17] reported that corona discharge could reduce the concentration of HCHO from 0.8 ppm to 0 ppm in 13 min. An ESP is commonly paired with a downstream catalyst to increase the HCHO removal rate and to reduce the amount of O_3_ generated [18,19].

ESPs generate O_3_ during the discharge process. Although O_3_ can oxidize and remove air pollutants, such as volatile organic compounds and bioaerosols [8,20], excessive O_3_ production causes secondary pollution. On the basis of the polarity of the discharge electrodes, ESPs can be classified as positive or negative corona ESPs. At the same operational voltage, negative corona ESPs have higher suspended particulate removal rates but also generate more O_3_ [21,22]. The O_3_ concentration produced by negative corona ESPs can reach five times that produced by positive corona ESPs [23,24]. The amount of O_3_ generated is also positively correlated with the ESP discharge voltage [25]. Generally speaking, the O_3_ concentration generated by positive corona ESPs is lower; thus, most commercial ESP air cleaners are positive corona ESPs. In the air, O_3_ easily forms unstable free radicals, which can destroy the external membranes of cells, resulting in the leakage of cytoplasm and effectively killing bacteria and fungi [20,26,27].

Many studies have confirmed that ESP has a high efficiency in removing suspended particulates. However, for the removal of gaseous pollutants and bioaerosols, other equipment (such as activated carbon filter or UVGI, etc.) is usually used instead, and the potential of gaseous pollutants and bioaerosols removal of ESP is not well identified. Therefore, this study focuses on the impact of negative discharge on air pollution and the contribution of O_3_ in the process of removing air pollutants. In this study, the air pollutant removal performance of a negative corona ESP was explored. Performance was measured for removal of suspended particulates (PM_2.5_ and PM_10_), formaldehyde (HCHO), and bioaerosols (bacteria and fungi). The O_3_ concentration generated during the ESP discharging process was also measured. In the experiment, ESP performance was first investigated in the laboratory. Next, ESP was placed in field test environments (a food waste storage room and a meeting room) to assess its actual air pollutant removal performance.

## 2. Materials and Methods

### 2.1. Experimental Apparatus and Analysis Methods

**Figure 1:**
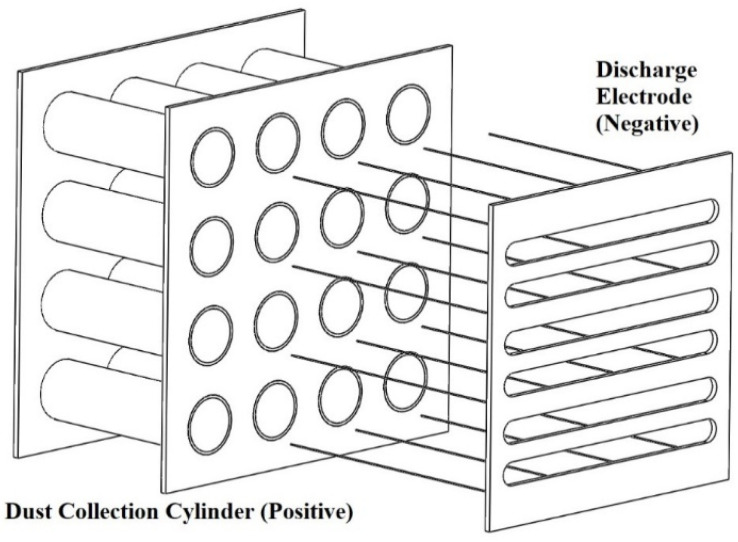
Side schematic view of electrode module in ESP.

In this study, we randomly selected ESP air cleaner on the market without qualifications. A negative corona ESP air cleaner was used. The negative corona discharge electrode modules were wire-to-cylinder structures, and the collection plate consists of 136 groups of cylinders with a radius of 0.75 cm and a height of 2 cm (Figure 1), and the dust collection plate was multiple sets of cylindrical structures with an effective dust collection area of 0.13 m^2^. The ESP inlet and outlet areas were both 0.04 m^2^. The voltage of the ESP could be set to a low (−6 kV) or high (−10.5 kV) negative voltage. The operational air flow rate could be set to low (2.4 m^3^/min, 0.04 m^3^/s) or high (4.8 m^3^/min, 0.08 m^3^/s).

The concentrations of five air pollutants were measured, namely suspended particulates (PM_2.5_ and PM_10_), HCHO, bioaerosols (bacteria and fungi), and O_3_. Table 1 presents the parameters of the experimental devices. A device was used to measure the real-time concentration of air pollutants at the ESP inlet (C_in_) and outlet (C_out_). For each experiment, at least five groups of data were recorded consecutively. The measurement duration for each group of data was 2–5 min. The mean concentration of the measurement data of C_in_ and C_out_ at the steady state was then calculated. Every experiment involved two repeated tests, and the difference between the mean values from the two experiments was within ±10% except for the difference between the mean bioaerosol values, which was within ±20%. Finally, the mean concentrations of air pollutants (C_in_ and C_out_) were used to calculate ESP pollutant removal efficiency (Equation (1)).

(1) $$\mathrm{Removal}\;\mathrm{efficiency}\;(\%)=\left[\frac{\left(C_{\mathrm{in}\;}{-\;C}_{\mathrm{out}}\right)}{C_{\mathrm{in}}}\right]\;\times \;100\%$$

The bioaerosols (bacteria and fungi) sample collection and cultivation methods adopted in this study were in accordance with the standard method for analyzing bacteria and fungi concentrations in air (NIEA E301.12C and NIEA E401.12C [28,29]) announced by the Environmental Protection Administration of Taiwan. To collect bacteria and fungi samples, an Anderson two-stage sampler was utilized to collect strains and spores in the air. Petri dishes with the collected samples were sent to the laboratory for cultivation. Bacteria and fungi were cultivated at 30 °C for 48 ± 1 h and at 25 °C for 96 ± 12 h, respectively. After cultivation, the colony counts of the bacteria and fungi samples were calculated. The total colony count in the air in the sample was then calculated with Equation (2). For bacteria and fungi sampling, at least two repeat tests were conducted. Before sampling, a flow calibrator was used to calibrate the flow of the Andersen two-stage sampler. The difference between the flow before and after sampling was set to be smaller than ±10%. The limit of detection (LOD) of the bacteria and fungi sampling method was determined in accordance with the analysis method disclosed by the Environmental Protection Administration of Taiwan (Equation (3)).

(2) $$\mathrm{Bioaerosol}\;\mathrm{Conc}.(\frac{\mathrm{CFU}}{m^{3}})=\frac{\mathrm{Colony}\;\mathrm{forming}\;\mathrm{unit}\left(\mathrm{CFU}\right)}{\mathrm{Flow}\;28.3\left(\frac{L}{\min}\right)\times \mathrm{Time}\;\mathrm{Sampling}\;\left(\min\right)}\times 1000(\frac{L}{m^{3}})$$

(3) $$\mathrm{LOD}=\frac{1000}{28.3\times 2}≦18\frac{\mathrm{CFU}}{m^{3}}$$

On the basis of the size of the particles, the charging effect of ESP on particles can be classified as having two mechanisms: diffusion charging and field charging [30]. Before calculating the theoretical particle removal efficiency, the unit electric charge that a particle could obtain was calculated for diffusion charging and for field charging by using Equations (4) and (5), respectively. Equation (6) was then used to calculate the terminal velocity of particles in ESP.

(4) $$n=\frac{\mathrm{DkT}}{{2K}_{E}e^{2}}\ln\;[1+\frac{{\pi K}_{E}{\mathrm{Dc}}_{i}e^{2}N_{i}t}{2\mathrm{kT}}]$$

(5) $$n=\frac{3\varepsilon }{\varepsilon +2}\left[\frac{{\mathrm{ED}}^{2}}{{4K}_{E}e}\right][\frac{{\pi K}_{E}{\mathrm{eZ}}_{i}{\mathrm{tN}}_{i}}{{1+\pi K}_{E}{\mathrm{eZ}}_{i}{\mathrm{tN}}_{i}}]$$

(6) $$V=\frac{{\mathrm{neEC}}_{c}}{3\pi \mu D}$$

Here, *n* is the charge of a particle (C), D is the diameter of the particle (μm), T is the absolute temperature (K), k is the Boltzmann constant (N∙m/k), K_E_ is the electrostatic constant (N∙m^2^/C^2^), e is the charge of the electron (C), C_i_ is the mean thermal motion of radicals (m/s), N_i_ is the ion concentration (radicals/m^3^), *t* is the residence time (s), ε is the dielectric constant of the particle (C^2^/N∙m^2^), E is the electric field strength (V/m), Z_i_ is the electric mobility of radicals (m^2^/V∙s), V is the terminal velocity (m/s), µ is the viscosity coefficient of gas (N∙s/m^2^), and C_c_ is the slip coefficient.

After obtaining the terminal velocity of the particles in ESP, the Deutsch–Anderson equation (Deutsch, 1992) [31] was used to calculate the theoretical removal rate. ɳ is the total removal rate (%) under the two mechanisms of diffusion charging and field charging, A is the effective area of the ESP dust collection plate (m^2^), V is the terminal static velocity (m/s), and Q is the inflow air flow rate of the ESP (m/s).

(7) $$ɳ=\left[1-\exp\left(-\frac{\mathrm{AV}}{Q}\right)\right]\times 100\%$$

### 2.2. Laboratory Test System

**Figure 2:**
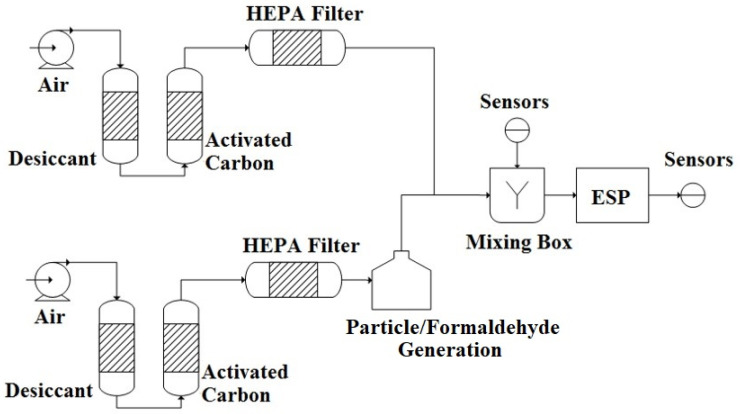
ESP of lab test system.

In the laboratory test system (Figure 2), the temperature and relative humidity (RH) were maintained at 24 ± 1 °C and 55 ± 5% RH, respectively. PM_2.5_, PM_10_, and HCHO pollutant concentrations were measured. An AGK-2000 suspended particle generator was used to generate the suspended particulates. NaCl(ap) was atomized and then dried to generate the required PM_2.5_ and PM_10_ concentrations. Particulates were input to the AGK-2000 through a mixing chamber; dry air (30–50% RH) filtered using activated carbon was input to the mixing chamber and could be used to adjust the concentration of the suspended particulates in the chamber. The standard concentrations listed in Taiwan’s Indoor Air Quality Act (PM_2.5_: 35 μg/m^3^∙24 h; PM_10_: 75 μg/m^3^∙24 h) were used as a reference; substantially larger concentrations were used as the experimental concentrations (70 ± 10 μg/m^3^ and 150 ± 20 μg/m^3^, respectively.) After the suspended particulates passed through the mixing chamber and entered the ESP, a device was used to measure the concentrations of PM_2.5_ and PM_10_ in the mixing chamber and at the outlet of the ESP. For the HCHO experiment, paraformaldehyde particles were used to prepare an HCHO solution. High-pressure air filtered with activated carbon was mixed into the HCHO solution, and HCHO was atomized with the aeration atomization method. HCHO gas was added to the mixing chamber and mixed with dry air that had been filtered with activated carbon. In the experimental setting, HCHO’s concentration was 0.400 ± 0.010 ppm (the concentration listed in the Indoor Air Quality Act is 0.08 ppm). HCHO gas then entered ESP. A device was used to measure the concentration of HCHO in the mixing chamber and at the outlet of the ESP.

In the experiment investigating O_3_ concentrations, we first conducted the O_3_ background concentration experiment for ESP. In the laboratory testing system, dry air (at 55% RH) that had been filtered with activated carbon and had no added pollutants was fed into ESP. The O_3_ concentrations at the ESP inlet and outlet were measured. Next, O_3_ experiments with suspended particulates were conducted. The aerosol generator was used to generate air with a mean concentration of 1081 µg/m^3^ PM_2.5_ and 2601 µg/m^3^ PM10, which was inputted to ESP. The O_3_ concentrations were measured at the inlet and outlet.

### 2.3. Environmental Conditions for Field Tests

**Figure 3:**
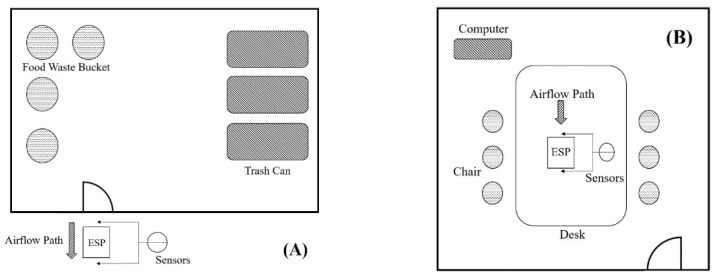
Site plan of the field test on (**A**) food waste storage room and (**B**) meeting room.

Field tests were performed in a food waste storage room at a university cafeteria and in a university meeting room. The concentrations of environmental pollutants are presented in Table 2. The food waste storage room had an area of 15.8 m^2^ and height of 2.5 m. When the experiment was conducted, the indoor temperature and humidity were 26.0 °C and 85% RH, respectively. The food waste storage room was in the basement. The room is humid and warm; thus, it was suitable for the growth of bacteria and fungi. The door to the food waste storage room was kept open. ESP was placed outside the door of the food waste storage room, and its inlet was oriented toward the food waste storage room. Figure 3A presents the layout of the food waste storage room and ESP’s location. Concentrations of PM_2.5_, PM_10_, HCHO, and bioaerosols (bacteria and fungi) were measured.

The meeting room had an area of 4.5 m^2^ and height of 3 m. When the experiment was conducted, indoor temperature and humidity were 23.5 °C and 70% RH, respectively. During the experiment, the air conditioning was not switched on, and the doors and windows were closed. Figure 3B presents the placement of the ESP. Concentrations of bioaerosols (bacteria and fungi) were measured.

## 3. Results

### 3.1. Assessing ESP Removal Performance for Aerosols

**Figure 4:**
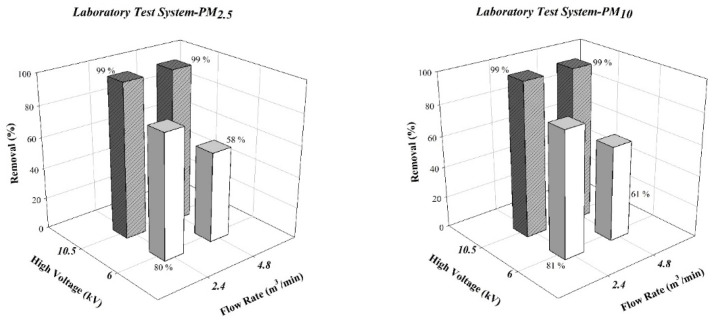
Experimental results of particle in the laboratory test system.

The calculation results of the Deutsch–Anderson equation revealed that, at a discharge voltage of −6 kV and air flow rate of 2.4 m^3^/min, the theoretical PM_2.5_ and PM_10_ removal rates were 80% and 51%, respectively; at 4.8 m^3^/min, these rates were 81% and 43%, respectively. At −10 kV, the theoretical PM_2.5_ and PM_10_ removal rates were 99% at both air flow rates. The results for the laboratory test system are presented in Figure 4. At a discharge voltage was −6 kV, increasing the air flow rate from 2.4 m^3^/min to 4.8 m^3^/min reduced the PM_2.5_ and PM_10_ removal rates from 80% to 58% and from 81% to 61%, respectively. If the ESP discharge voltage was −10 kV, the PM_2.5_ and PM_10_ removal rates were both 99% at both air flow rates. The experimental results for the laboratory test system and the theoretical PM_2.5_ and PM_10_ removal rates were in good agreement. Moreover, at a high discharge voltage, ESP had excellent removal efficiency for aerosols.

**Figure 5:**
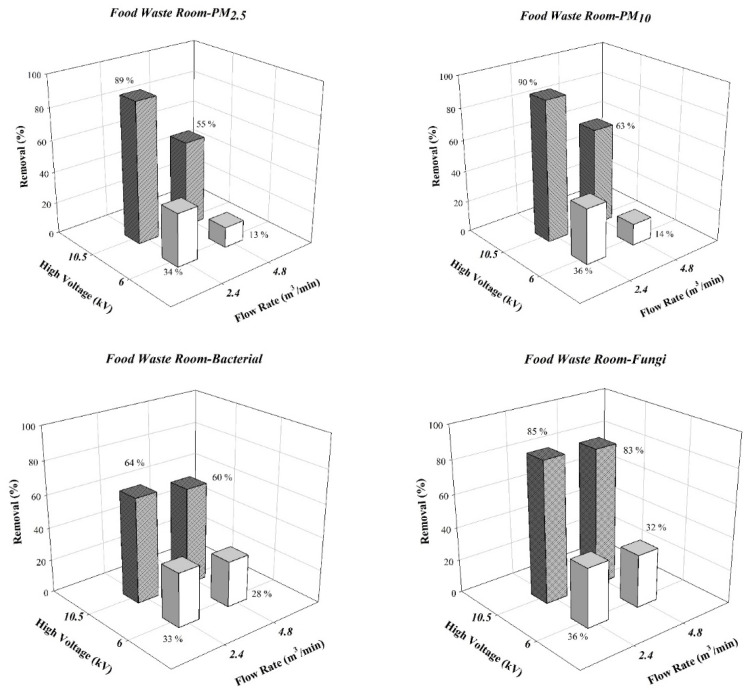
Experimental results of particle and bioaerosols in the food waste storage room.

**Figure 6:**
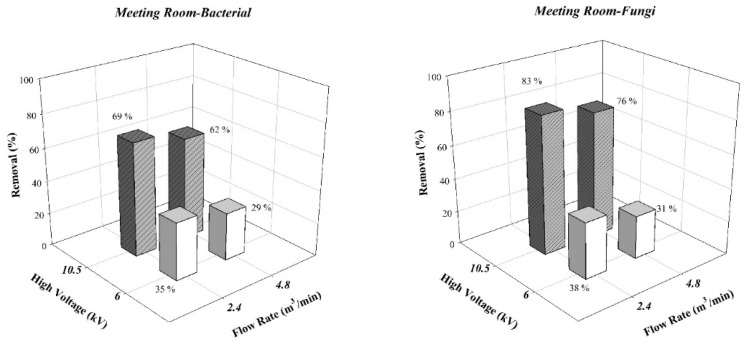
Experimental results of bioaerosols in the meeting room.

However, reduced ESP aerosol particle removal rates were observed in the field tests. Figure 5 reveals the experimental results for the food waste storage room. If the ESP discharge voltage was −10 kV, PM_2.5_ and PM_10_ removal rates were 89% and 90%, respectively; those for bacteria and fungi were 64% and 85%, respectively. Figure 6 presents the experimental results for the meeting room. The bacteria and fungi removal rates reached 69% and 83%, respectively. The Deutsch–Anderson equation (Equation (7)) was used to calculate theoretical removal rates; the environmental temperature both affects the gas viscosity and reduces particle terminal velocity. However, the temperatures in the laboratory (24 °C) and in the field (23.5–26 °C) did not differ substantially. We inferred that the humidity of the environment was the key reason for the differences in removal efficiencies. In the laboratory test system, the relative humidity was controlled at 55% RH, whereas that at the food waste storage room was at 85% RH. Air flow with high humidity in ESP affected both the strength of the electric field and the size of the ESP corona [32]. Wang and You (2013) [33] discovered that if the humidity of air in the reaction chamber of ESP increased, water molecules were ionized by the corona and agglomerated into water ion groups. Although this phenomenon increased the total number of electric charges in the electric filed, the water ion groups gathered near the discharge electrode and thus reduced the size of the corona field and hindered the electron avalanche process.

The results for suspended particulates and bioaerosols both revealed that discharge voltage had a greater effect on the removal rate than the operational air flow rate of the ESP. If the discharge voltage increased from −6 kV to −10.5 kV, the suspended particulate and bioaerosol removal rates both increased. The experimental results of the field tests (Figure 5 and Figure 6) revealed that PM_2.5_ removal rates increased from 13–36% to 55–89%, and PM_10_ removal rates increased from 14–36% to 63–90%. The bioaerosol removal rates in the food waste storage room increased from 28–36% to 60–85%, and those in the meeting room increased from 29–38% to 62–83%. Kawada et al. (2002) [34] suggested that an increase in ESP discharge voltage resulted in an increase in its particle removal rate. At a high discharge voltage, ESP generates a stronger and broader corona field, thus increasing charging efficiencies for particles. With the charging voltage held constant, an increase in the air flow rate from low (2.4 m^3^/min) to high (4.8 m^3^/min) reduced the removal rates for both suspended particulates and bioaerosols. If the discharge voltage of the ESP was fixed at −10 kV and the air flow rate was adjusted from 2.4 m^3^/min to 4.8 m^3^/min, the optimal suspended particulate removal rate in the food waste storage room was reduced from 89–90% to 55–63%, and the optimal bioaerosol removal rate was reduced from 64–85% to 60–83%. The same trend was observed in the meeting room; the optimal bioaerosol removal rates were reduced from 69–83% to 61–76%. The Deutsch–Anderson equation reveals that if the air flow rate increases, the removal rate decreases because air remains in ESP for a shorter duration, decreasing the charging time for particles in the electric field and the duration that the charged particles can be attracted by the dust collection plate. Thus, particles have an increased probability of passing through the dust collection plate [35].

The bioaerosol experimental results revealed that the bacteria and fungi removal rates in the field tests were both lower than the theoretical removal rates calculated for PM_2.5_ and PM_10_. Bioaerosols typically have aerodynamic diameters between 0.1 and 30 µm [36,37]. Analyses of different bacteria were not conducted. Thus, we assumed that most bioaerosols in the field tests had a particle diameter sufficient to become charged by the electric field (i.e., aerodynamic diameter > 1 µm). Fungal spores have larger particle diameters (1–30 μm) than bacteria (0.25–8 μm) [38]. Thus, the bacteria and fungi removal rates were compared with theoretical PM_2.5_ and PM_10_ removal rates, respectively. According to the Deutsch–Anderson equation [31], if the particle diameter is large, the removal rate is high. The field experimental results also revealed that the fungi removal rate was higher than that for bacteria. Bioaerosols of 0.1–1 µm are at a transitional size between being affected by field charging and diffusion charging (aerodynamic diameters < 1 µm); thus, they were charged inefficiently, resulting in a reduced removal rate. Field test results also revealed that the bacteria and fungi removal rates were both lower than PM_2.5_ and PM_10_ removal rates. We attributed this result to the presence of bioaerosols with electrical resistance in the food waste storage room, resulting in differences between theoretical and experimental PM_2.5_ and PM_10_ removal rates. If the outer layers of a microorganisms have the same polarity as the discharge electrode (and thus have electrical resistance), these microorganisms are less likely to be removed by an ESP. Moreover, these bioaerosols affect the charging of other particles in the scope of the corona [39]. The surfaces of airborne microorganisms contain several chemical substances that can be ionized, such as proteins, amino groups (–NH_2_), and carboxyl groups (–COOH). These cause microorganisms to carry charges in their natural state. However, charges on microorganisms also have other origins and further complexity. If a bioaerosol is covered by a liquid droplet, it may carry both positive and negative charges. When the droplet dries, these charges are transferred to bioaerosols; if the charges are the same or opposite polarity as the bioaerosols, the bioaerosols will carry higher or lower charge, respectively [40]. The total charge carried by bioaerosols entering the ESP comprises not only the charge carried by the microorganisms themselves but also the charge obtained due to charging from the ESP’s electric field. Together, these increase the probability of bioaerosols being collected by the dust collection plate. If the ESP’s corona discharge was high (−10 kV), a favorable bioaerosol removal rate was observed. This result was in accordance with that of Mainelis et al. (2002) [41].

However, bioaerosols differ in the amount of charge that they carry and their polarities. For example, *Escherichia coli* carries a relatively high positive charge compared with many other bacteria [42]. In negative corona ESP, the charge carried by *E.* *coli* is neutralized by the electric field; the bacteria cannot easily be collected by positively charged dust collection plates. However, even if the charge carried by bioaerosols is the opposite polarity of the ESP discharge, the amount of charge generated by corona discharge is typically far greater than that carried by bioaerosols. Mainelis et al. (2002) [43] revealed that completely removing the charge of bioaerosols is challenging. If the charge on bioaerosols is weakened, they could still be collected by the dust collection plate; however, the removal rate would be lower.

For an ESP discharge voltage of −10 kV and a high air flow rate (4.8 m^3^/min), the bioaerosol removal rates in the food waste storage room and the meeting room were both higher than PM_2.5_ and PM_10_ removal rates in the food waste storage room. If the air flow rate of the ESP was adjusted, the difference in bioaerosol removal rate was small, indicating that mechanisms other than the dust collection plate contributed to the removal of bioaerosols. The high-concentration O_3_ generated by corona could destroy microorganisms. However, in an environment with high humidity, minute droplets coagulate on and cover the surface of microorganisms, increasing the particle diameter of the bioaerosols [44]. This phenomenon reduces the rate of successfully charging bioaerosols in the electric field. Moreover, these droplets protect the microorganism by decreasing the likelihood of their destruction in the electric field. This is from the experimental results in which corona has a removal effect on bioaerosol, although higher ambient humidity will reduce the air pollutant removal efficiency of ESP. However, it can still be observed that ESP can destroy parts of the bioaerosol.

### 3.2. ESP Ozone Generation and Its Potential for Air Pollutant Removal

**Figure 7:**
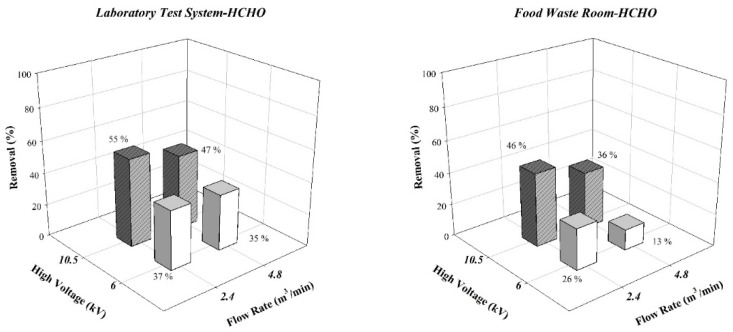
Experimental results of the formaldehyde.

Figure 7 presents the results for the removal of HCHO with an ESP in the laboratory test system and food waste storage room. At the same discharge voltage, higher air flow rates resulted in the air remaining in the ESP for a reduced duration, leading to a reduced rate of HCHO removal. At the same amount air flow rate, a stronger discharge intensity led to an increased removal rate. If the discharge voltage was fixed at −10.5 kV low (2.4 m^3^/min) and high (4.8 m^3^/min), air flow rates achieved single-pass HCHO removal rates of 55% and 47%, respectively, in the lab tests and 46% and 36% in the waste storage room, respectively.

Chang et al. (1995) [45] reported two mechanisms for the removal of airborne HCHO by the corona: (1) The electrons emitted from the discharge electrode directly collide with the HCHO molecules and break its chemical bonds. (2) Corona discharge turns other gas molecules in the air into free radicals. Free radicals then generate chemical reactions with HCHO, turning HCHO into more stable substances such as CO, CO_2_, or H_2_O. In the electric field, HCHO is decomposed into the formyl radical (–CHO). This step is critical; by applying an electric field with suitable strength, the bond (4.3 eV) between H and C can be broken, decomposing HCHO into –CHO and H^+^ [46]. In addition to corona destruction, a strong electric field ionizes numerous substances. Among these, OH^−^ is a major contributor for oxidizing HCHO. Lu et al. (2012) [47] discovered that airborne H_2_O_(g)_ was dissociated into OH^−^ and H^+^ by corona. At an appropriate environmental humidity of 37% RH, an optimal HCHO removal rate (≈65%) was observed.

During ESP corona discharge, in addition to using a high-energy electric field to destroy HCHO and bioaerosols, the generated O_3_ can also oxidize and decompose HCHO and bioaerosols. If the electrons in the ESP corona discharge collide with oxygen molecules in the corona layer and form atomic oxygen, atomic oxygen further reacts with other oxygen molecules and forms O_3_ [48]. The negative corona ESP used in this study generated an enormous amount of O_3_. To understand the changes in O_3_ concentrations during ESP operation, the amount of O_3_ generated was measured in the laboratory system. In the ozone-background test, no pollutants were added. Dry air filtered with an activated carbon filter was input to the ESP, and the O_3_ concentration at the ESP outlet was measured. For the ozone-suspended particulate test, suspended particulates were input to the ESP, and the O_3_ concentration at the ESP outlet was measured. The environmental background concentration of O_3_ must be subtracted from that measured at the ESP outlet. Thus, the O_3_ concentrations were normalized by measuring the initial background O_3_ concentration at the inlet of the ESP (0.011 ± 0.002 ppm) and substracting this value from the values at the outlet. Table 3 lists the experimental results. For the ozone-background and test, the O_3_ concentration reached 7.429 ppm. The O_3_ concentration generated by the ESP was slightly lower in the ozone-suspended particulate test. The discharge voltage and air flow rate both affected the generated O_3_ concentration. A high ESP discharge voltage increases the strength of the electric field and expands the corona, increasing the probability of ionizing the air and resulting in the generation of a higher O_3_ concentration [22,49,50]. If the discharge voltage was constant, an increase in the air flow rate increased the speed of the gas molecules in the chamber. Thus, the time for the molecules to be ionized to form O_3_ was reduced, leading to a reduction in the amount of generated O_3_ [51].

**Figure 8:**
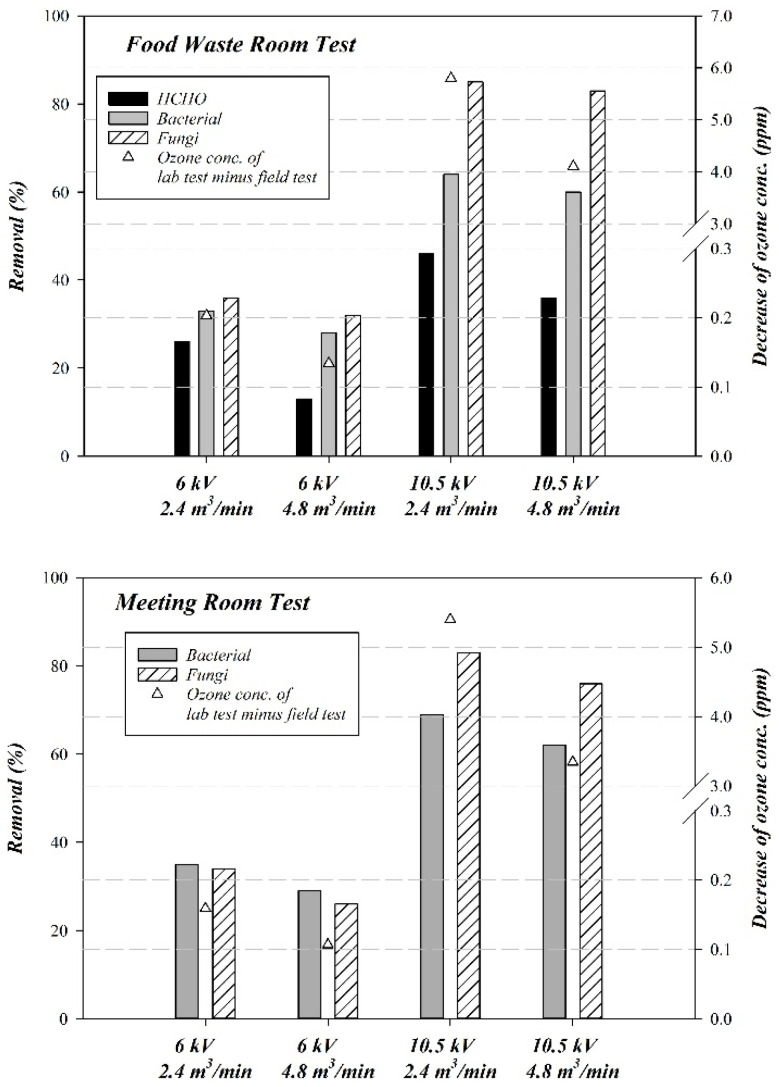
Comparing ozone concentration and air pollutant removal efficiency.

In the food waste storage room and the meeting room, the background O_3_ concentrations measured at the ESP inlet were 0.23 ppm and 0.01 ppm, respectively. The O_3_ background concentration was higher in the food waste storage room than in the meeting room because the storage room had ultraviolet (UV) germicidal lamps (254 nm). Shortwave UV light (100–280 nm) destroys gaseous O_2_, forming unstable O that rapidly reacts with other O_2_ molecules to form O_3_ [52,53]. Table 3 reveals that the O_3_ concentrations measured at the ESP outlet in the field tests were lower than those measured in the ozone-background test in the laboratory test system. The increase in O_3_ concentration at the outlet in the field was lower than that in the laboratory system, and the difference increased as the discharge voltage increased (Figure 8). Under the experimental conditions of high discharge voltage (−10.5 kV) and low air flow rate (2.4 m^3^/min), the difference in the O_3_ concentrations was the largest.

The smaller increase in O_3_ concentrations in the field compared with that in the laboratory system was attributed to two phenomena: (1) The O_3_ generated during the discharge process reacts with air pollutants inside the ESP. (2) The generation of O_3_ in the ESP is hindered by humidity or other environmental factors. For the O_3_ tests in the laboratory system, the air input to the ESP was first manipulated to obtain 55% RH in the mixing chamber. By contrast, RH in the food waste storage room (85%) and the meeting room (70%) could not be controlled. Several studies have reported that RH in the environment substantially affects the corona discharge of ESP [32,33]. If RH is high, the amount of O_3_ generated by ESP is reduced [22,24]. If air with high humidity flows into the ESP, the electrons emitted by the discharge electrode collide with the water molecules, forming free radicals (OH^−^) and H_2_O_2_, both of which are extremely strong oxidizers. In addition, OH^−^ degenerates O_3_ to form HO_2_ and O_2_ [54,55]. Wang and Chen (2008) [56] stated that, in a humid environment, O_3_ generated by a negative corona ESP would be decomposed. Moreover, the generation rate of O_3_ is reduced at high humidity. At 0 RH%, the concentration and generation rates of O_3_ were 13 ppm and 6.1 × 10^−3^ mg/m∙s, respectively. At 100 RH%, the concentration and generation rates of O_3_ were 3.1 ppm and 1.54 × 10^−3^ mg/m∙s, respectively.

O_3_ concentration is affected not only by the RH of the environment but also by reactions between O_3_ and airborne pollutants in the air and by O_3_ decomposition. Chang et al. (1995) [45] discovered that OH^−^ plays a key role in the reaction of O_3_ with airborne HCHO. The OH^−^ group binds with H^+^ in HCHO to produce the –CHO ion, which then reacts with other substances, such as OH^−^, H^+^, O^2−^, and O_2_, eventually becoming relatively harmless CO_2_ and H_2_O. However, these chemical reactions require sufficient energy. Fan et al. (2010) [57] stated that the reaction of O_3_ and HCHO is highly unfavorable without a catalyst and can be ignored. Moreover, in the absence of the electric field or if molecules cannot be converted to radicals (such as OH^−^ and O^2−^), the addition of catalysts does not trigger the reaction between O_3_ and HCHO. Although O_3_ can oxidize HCHO, it also reacts with OH^−^, resulting in a reduced removal rate of HCHO and bioaerosols [47]. This may be the reason that the HCHO removal rate in the laboratory system was higher than that in the food waste storage room. Moreover, the difference in O_3_ concentrations were assumed to be due to both environmental humidity and the reaction of O_3_ with OH^−^.

ESP removes bioaerosols not only through decomposition through the reaction with radicals (OH^−^) generated in the corona but also through damage caused by bombardment with high-speed electrons or radicals, breaking, or even penetrating microorganism outer membranes and entering to damage their DNA [58,59]. As a result, bioaerosols that are not collected by the dust collection plate cannot survive due to membrane damage causing cytoplasm leakage. Moreover, O_3_ is exceptionally effective for killing bacteria and fungi. Due to its strong oxidation ability, it can decompose microorganism cell membranes, resulting in cytoplasm leakage and death [27]. For bacterial species such as *Bacillus* that transform into endospores and become dormant, O_3_ can also effectively damage the endospore outer membrane, causing a loss of activity and death [20]. Dyas et al. (1983) [60] conducted experiments that revealed that an O_3_ concentration in a single-patient ward of 1 ppm could kill 95% of the bacteria and fungi in the ward. However, the concentration and contact time required for O_3_ to kill bioaerosols differed between species [61]. If the environment has relatively high humidity, water forms minute droplets that attach to the surface of bioaerosols, forming a liquid membrane. If O_3_ contacts these bioaerosols, it dissolved in the liquid membrane and is ineffective for killing bacteria or fungi. By contrast, Li and Wang (2003) [62] discovered that if the environmental humidity is high, more OH^−^ was generated and the efficiency for removing bioaerosols increased.

Because the differences in the amount of O_3_ in the food waste storage room and the meeting room were similar (Figure 8), an ESP discharge voltage of −10.5 kV resulted in optimal bacteria and fungi removal rates of 76–83%. We maintain that, although O_3_ can remove bioaerosols, corona and reactions with OH− were the primary reasons for their destruction. The removal of HCHO may be caused by similar pathways. However, other mechanisms that degrade or damage HCHO and bioaerosols cannot be eliminated. We can observe that ESP has removal effects on HCHO and bioaerosols, and the changing trend in removal efficiency potential is similar to PM_2.5_ and PM_10_.

Although O_3_ removes HCHO and bioaerosols, the O_3_ generated by an ESP can affect human health. We, therefore, advised adding activated carbon filters at the outlet of negative corona ESPs to effectively absorb O_3_ as well as any remaining gaseous pollutants. O_3_ may be removed by both its reaction with activated carbon and its absorption by activated carbon, resulting in decomposition [63]. Lee and Davidson (1999) [64] demonstrated that activated carbon had a favorable O_3_ removal rate; the initial removal rate reached 98%. Therefore, when selecting an ESP for indoor use, choosing the ESP with an activated carbon filter at the outlet is prioritized, which can avoid the situation of high O_3_ concentrations in the room.

## 4. Conclusions

Negative corona ESPs were used to effectively remove suspended particulates, formaldehyde, and bioaerosols. The performance of the ESP may be affected by environmental humidity. The pollutant removal rates at the field tests (70–85% RH) were all lower than that in the lab tests (55% RH). The optimal PM2.5, PM10, formaldehyde, bacteria, and fungi removal rates in the field tests reached 89%, 90%, 46%, 69%, and 85%, respectively. The results revealed that increasing the ESP discharge voltage increased its air pollutant removal rate. Reducing the air flow rate also increased the removal rate. At high voltage (−10.5 kV) and low air flow rate (2.4 m^3^/min), ESP achieved its optimal air pollutant removal rate.

If ESP was operated at a high discharge voltage, it had a favorable air pollutant removal rate; however, it generated a large amount of O_3_. At high voltage (−10.5 kV) and a low air flow rate (2.4 m^3^/min), the highest O_3_ concentration was observed at the ESP outlet. The experimental results for O_3_ and the pollutants revealed that the suspended particulates were not affected by the amount of O_3_ generated by ESP. Thus, we inferred that only a small proportion of the removed HCHO and bioaerosols were removed due to the oxidation and decomposition by O_3_; the majority was removed by corona destruction and reaction with OH^−^. However, the amount of O_3_ generated by the ESP is substantially affected by the RH in the environment. Consequently, the contribution of O_3_ for removing HCHO and bioaerosols could not be accurately evaluated.

The amount of O_3_ generated in the field tests was lower than that in the lab tests. This result was attributed to the effects of the relatively high RH on site and because O_3_ was consumed through reactions with air pollutants and radicals (such as OH^−^). Negative corona ESP had favorable removal performance for air pollutants, but it also generated high concentrations of O_3_. When using these devices, the risk of damage to the human body caused by high-concentration O_3_ cannot be ignored. For the problem that ESP may cause excessive indoor O_3_ concentration, it is recommended to choose an ESP with an activated carbon filter adsorption at the air outlet. In addition, while the negative corona ESP produces a higher concentration of O_3_, positive corona ESP can be used instead, which will reduce the amount of O_3_ produced, but pollution removal efficiency will also decrease. Although we believe that O_3_ and OH^-^ amounts are key factors for the direct destruction or oxidation of HCHO and bioaerosols, the contribution of O_3_ or OH^-^ has not been separately evaluated from particle collection. We expect that, in future studies, the role of ozone and oxidizing radicals in the corona discharge process can be quantitatively analyzed and that it can be further utilized by properly setting operation parameters.

## Figures and Tables

**Table 1:** Details of instruments for indoor air quality sampler.

Item	Instrument/Model	Principle	Detection Range	Resolution
PM_2.5_/PM_10_	AEROCET MetOne 531	Laser diode 5 MW, 780 nm	0.0001–1 mg/m^3^	0.5 μm
HCHO	PPM Technology/PPM Formaldmeter htv-m	Electrochemical	0.001–10 ppm	0.01 ppm
Bacteria/fungi	Thermo/Anderson two-stage sampler	Impacting on agar with incubation (Q: 28.3 LPM)	Stage 0 (8–24 μm) Stage 1 (1–8 μm)	-
O_3_	2B Model 202 Ozone Monitor	UV Absorption at 254 nm	1.5–100 ppb	0.1 ppb

**Table 2:** Background of concentration in lab and field tests.

	PM_2.5_ (μg/m^3^)	PM_10_ (μg/m^3^)	HCHO (ppm)	Bacterial (CFU/m^3^)	Fungi (CFU/m^3^)
Lab test system	70 ± 10	150 ± 20	0.400 ± 0.010	-	-
Food waste storage room	56 ± 39	94 ± 57	0.067 ± 0.027	176 ± 66	1388 ± 705
Meeting room	N.D. *	N.D. *	N.D. **	91 ± 45	213 ± 105

N.D. The concentration is below the limit of detection of instrument (* LOD: 1 μg/m^3^; ** LOD: 0.001 ppm).

**Table 3:** Ozone generated by lab system test and field test.

	6 kV		10 kV	
	2.4 m^3^/min	4.8 m^3^/min	2.4 m^3^/min	4.8 m^3^/min
Lab system ozone-background test				
Avg. Conc. (ppm)	0.229 ± 0.011	0.147 ± 0.011	7.148 ± 0.281	4.754 ± 0.029
Lab system ozone -particle test *				
Avg. Conc. (ppm)	0.181 ± 0.006	0.143 ± 0.011	7.374 ± 0.191	4.611 ± 0.028
Food waste storage room test				
Avg. Conc. (ppm)	0.026 ± 0.005	0.013 ± 0.003	1.347 ± 0.150	0.647 ± 0.041
Meeting room test				
Avg. Conc. (ppm)	0.070 ± 0.008	0.040 ± 0.004	1.749 ± 0.364	1.410 ± 0.424

* PM_2.5_: 1081 ± 72 µg/m^3^; PM_10_: 2601 ± 197 µg/m^3^.

## Data Availability

Not applicable.
